# Diabetes management during and after Ramadan among pregnant women in Saudi Arabia: exploring self-efficacy, self-care, and glycemic control

**DOI:** 10.3389/fnut.2025.1643107

**Published:** 2025-08-18

**Authors:** Omar Mohammed Alamri, António Raposo, Ayoub Ali Alshaikh, Hani A. Alfheeaid, Ali Mohammed Alamri, Mohammed Abdullah Alasmri, Nada Mohammed Alwubayran, Ahmed Khaled Shukri, Thamer Alslamah, Najla A. Albaridi, Md Faruque Ahmad, Abdullah Y. Otayf, Ariana Saraiva, Najim Z. Alshahrani

**Affiliations:** ^1^Department of Obstetrics and Gynecology, Abha Maternity and Children Hospital, Abha, Saudi Arabia; ^2^CBIOS (Research Center for Biosciences and Health Technologies), ECTS (School of Health Sciences and Technologies), Lusófona University, Lisboa, Portugal; ^3^Family and Community Medicine Department, King Khalid University, Abha, Saudi Arabia; ^4^Department of Food Science and Human Nutrition, College of Agriculture and Food, Qassim University, Buraydah, Saudi Arabia; ^5^Aseer Health Cluster, Abha, Saudi Arabia; ^6^Department of Health Services and Hospital Administration, King Khalid University, Abha, Saudi Arabia; ^7^Abha Maternity and Children Hospital, Abha, Saudi Arabia; ^8^Department of Family and Community Medicine, College of Medicine, University of Jeddah, Jeddah, Saudi Arabia; ^9^Department of Public Health, College of Applied Medical Sciences, Qassim University, Buraydah, Saudi Arabia; ^10^Department of Health Science, College of Health and Rehabilitation, Princess Nourah bint Abdulrahman University, Riyadh, Saudi Arabia; ^11^Department of Clinical Nutrition, College of Nursing and Health Sciences, Jazan University, Jazan, Saudi Arabia; ^12^Research in Veterinary Medicine (I-MVET), Faculty of Veterinary Medicine, Lisbon University Centre, Lusófona University, Lisboa, Portugal; ^13^Veterinary and Animal Research Centre (CECAV), Faculty of Veterinary Medicine, Lisbon University Centre, Lusófona University, Lisboa, Portugal

**Keywords:** diabetes management, gestational diabetes mellitus, self-care, self-efficacy, pregnant women, Ramadan

## Abstract

**Background:**

Fasting during Ramadan poses distinct challenges for individuals with diabetes, especially pregnant women, due to increased metabolic demands and the heightened risk of hypoglycemia, hyperglycemia, and dehydration. Although medical guidelines often advise against fasting in this group, many women still choose to observe the fast. This study aims to explore diabetes self-efficacy and self-care behaviors during and after Ramadan among pregnant women.

**Methods:**

This cross-sectional study was conducted during and after Ramadan 2025 at Abha Maternity Hospital in Aseer region in Saudi Arabia. Pregnant women with gestational or pre-existing diabetes were recruited. Data were collected using validated Arabic versions of the Diabetes Self-Efficacy Scale and the Summary of Diabetes Self-Care Activities questionnaire. Paired t-tests and correlation analyses were used to examine changes and relationships.

**Results:**

A total of 162 pregnant women with diabetes participated in this study. Self-efficacy scores remained stable during and after Ramadan (mean 4.8 vs. 4.9, *p* = 0.2), while self-care scores declined significantly post-Ramadan (32.5 to 29.8, *p* = 0.001). HbA1c distribution shifted slightly, with fewer participants in the <5.7% range after Ramadan and more in the 5.7–6.4% range. Most managed diabetes using oral drugs with diet and exercise (42%). Medical education was linked to higher self-care scores (*p* < 0.001), while trial fasting improved self-efficacy (*p* = 0.001). Perceived glycemic control improved post-Ramadan (difficulty decreased from 72.8 to 65.4%, p = 0.001). Hypoglycemia caused 26.5% of fast-breaking episodes, though “other reasons” were more common. Older age and higher pregnancy order correlated with lower self-care and self-efficacy, while education and perceived health showed positive correlation.

**Conclusion:**

This study underscores the need for culturally sensitive, pregnancy-specific interventions to support safe fasting. Sustained education, individualized care, and preparation before Ramadan were linked to better outcomes, highlighting the importance of ongoing support beyond the fasting period for optimal diabetes management.

## 1 Introduction

Ramadan is a holy month of fasting observed by Muslims worldwide, during which individuals abstain from food, drink, and other physical needs from dawn until sunset (1). This period of spiritual reflection and devotion presents unique challenges for individuals with diabetes, especially pregnant women who are navigating both metabolic changes and the demands of gestation (2). Although Islamic teachings provide exemptions from fasting for those with medical conditions, many individuals with diabetes still choose to fast, underscoring the need for tailored medical guidance and culturally sensitive healthcare support (3).

Fasting during Ramadan alters the typical metabolic rhythm, potentially leading to complications such as hypoglycemia, hyperglycemia, dehydration, and thrombosis (2). These risks are amplified in pregnant women, who already experience insulin resistance and fluctuating glucose levels due to hormonal changes (4). Clinical guidelines recommend pre-Ramadan assessments 1–2 months in advance to evaluate an individual’s ability to fast safely (5). These evaluations typically include risk stratification, medication adjustments, education on blood glucose monitoring, and individualized care planning (2, 5). However, despite these guidelines, there remains a gap in research specific to pregnant women with diabetes, particularly in Middle Eastern contexts such as Saudi Arabia.

Emerging evidence supports the use of newer antidiabetic agents such as GLP-1 receptor agonists and DPP-4 inhibitors during Ramadan, as they are associated with a lower risk of hypoglycemia and improved glycemic outcomes compared to traditional sulfonylureas (5, 6). In addition, structured educational interventions and regular blood glucose monitoring have been shown to significantly enhance diabetes self-management during fasting (7). However, access to continuous glucose monitoring and individualized care remains limited in many healthcare settings, particularly for women who are managing both pregnancy and diabetes.

A recent cross-cultural study comparing individuals with diabetes in Saudi Arabia and Pakistan during Ramadan found that while many participants maintained glycemic control, a notable proportion still experienced hypoglycemic episodes (3). Pre-Ramadan physician visits and education were associated with safer fasting practices, though lifestyle challenges persisted during festive periods such as Eid-al-Fitr (3).

Given the physiological complexities of pregnancy and the cultural importance of fasting, more targeted research is needed to understand how pregnant women in Saudi Arabia manage their diabetes during and after Ramadan. This study seeks to explore factors such as self-efficacy, self-care behaviors, glycemic control, and the impact of medical advice and education. The findings aim to inform culturally responsive healthcare practices that support pregnant women in making safe, informed decisions about fasting during Ramadan.

## 2 Methods

### 2.1 Study design and setting

This cross-sectional study was conducted to explore factors impacting diabetes management during and after Ramadan among Saudi pregnant women in the Aseer region. The study employed a descriptive correlational and analytic design to examine the relationships between diabetes self-efficacy, self-management behaviors, and self-care activities in this specific population during the holy month of Ramadan.

### 2.2 Study population and sampling

The target population consisted of pregnant women diagnosed with diabetes (gestational diabetes mellitus or pre-existing diabetes) residing in the Aseer region of Saudi Arabia. Participants were recruited from the Tertiary hospital “Abha Maternity Hospital,” a specialized hospital in Gynecology in the Aseer region, during the 2025 Ramadan period and continued until May 2025.

### 2.3 Inclusion criteria

Pregnant women aged 18 years and above, diagnosed with gestational diabetes mellitus or pre-existing diabetes, residents of Saudi Arabia, able to read and understand Arabic, and willing to provide informed consent to participate during and after Ramadan, were included in the study.

### 2.4 Exclusion criteria

Pregnant women with severe pregnancy complications, those with cognitive impairments that would prevent completion of questionnaires, non-Saudi participants, and those who refused to participate in the study and did not complete the participation before and after Ramadan, were excluded.

### 2.5 Sample size calculation

The sample size was calculated using Medcalc 15.8. The primary outcome of interest is the percentage of DM in pregnant women in Saudi Arabia. A meta-analysis of gestational diabetes mellitus in the Eastern Mediterranean region revealed that the prevalence of gestational diabetes mellitus in Saudi Arabia was 11.7% (8). Based on this study, with an alpha error of 5%, study power of 80, and 5% precision with a design effect of 1. The calculated sample size is 162 participants.

### 2.6 Data collection

Data collection was conducted using structured self-administered questionnaires. The data collection period was during and after Ramadan till May 2025 to capture experiences both during and after Ramadan. Participants were recruited as they presented to maternal health clinics for routine prenatal appointments. Upon arrival at the clinic, potential participants were identified by healthcare staff based on the inclusion criteria. Research assistants approached eligible pregnant women in the waiting areas and provided them with detailed information about the study objectives, procedures, and their rights as participants.

After explaining the study purpose and addressing any questions or concerns, written informed consent was obtained from all participants who agreed to participate. The consent process ensured that participants fully understood their voluntary participation, the right to withdraw at any time, and the confidentiality measures in place to protect their personal information.

During the initial visit, a follow-up appointment was scheduled with each participant to complete the post-Ramadan assessment. Participants were contacted 3–5 weeks after the conclusion of Ramadan to arrange the follow-up data collection session. The follow-up assessment involved re-administration of the same instruments to evaluate any changes in diabetes self-efficacy and self-care activities following the Ramadan period.

### 2.7 Study instruments

#### 2.7.1 Demographic and clinical data questionnaire

A structured questionnaire was used to collect demographic and clinical information, including age, educational level, employment status, type of diabetes, residence, Ramadan fasting practices, diabetes before pregnancy, and ever diagnosed with DM during this pregnancy or any previous pregnancies.

#### 2.7.2 Arabic diabetes self-efficacy scale

The Diabetes Self-Efficacy Scale used in this study was developed by the Stanford Patient Education Research Center based on the Chronic Disease Self-Efficacy Questionnaire (9). The DSES assesses individuals’ confidence in diabetes self-management skills, including diet, physical activity, blood glucose monitoring, follow-up visits, and self-control. The scale comprises eight items answered on a Likert scale, with higher scores indicating higher diabetes self-efficacy. The reliability of the DSES was demonstrated with a Cronbach’s alpha of 0.85 when tested on 186 participants with diabetes (10).

#### 2.7.3 Summary of diabetes self-care activities questionnaire

The SDSCA questionnaire, developed by Toobert et al., was used to assess diabetes self-care behaviors in the Arabic Language (11). The questionnaire consists of two parts. The first part contains 11 items about self-care activities distributed across four subscales: diet (four items), exercise (two items), blood glucose testing (two items), foot care (two items), and smoking (one item). For this study, 10 items were included (excluding the smoking question), where respondents indicated how many days in the past 7 days, they performed specific self-care activities. This section has undergone extensive reliability and validity testing (12). The second part of the SDSCA covers several subscales exploring healthcare provider interventions regarding diet, exercise, blood glucose testing, and medication. However, this section has not been formally validated and was not included in the current study analysis.

The SDSCA instrument is widely used in clinical practice and diabetes-related research and has been successfully translated and validated in multiple languages.

### 2.8 Educational intervention

Pre-Ramadan medical education was delivered through structured group classes (60–90 min) combined with brief individual counseling sessions (20–30 min). The group sessions, conducted by certified diabetes educators and obstetricians, covered four key domains: (1) safe fasting practices, including recognition of warning signs; (2) self-monitoring of blood glucose with demonstration devices; (3) management of acute complications like hypoglycemia; and (4) tailored nutritional guidance for pregnancy. Individual sessions focused on medication adjustments and personalized risk assessment. All participants received standardized take-home materials (illustrated booklets and glucose logs) to reinforce learning. While the core curriculum was consistent across clinics, minor variations in delivery style occurred based on available staff and resources.

### 2.9 Ethical considerations

Ethical approval for this study was granted by the Research Ethics Committee at King Khalid University (Approval No. ECM#2024–1,105; HAPO-06-B-001). All participants provided written informed consent after receiving comprehensive information about the study’s objectives, procedures, potential risks, and benefits. Participation was entirely voluntary, with the right to withdraw at any time without penalty. Data confidentiality and participant anonymity were strictly maintained throughout the study.

### 2.10 Statistical analysis

Data were analyzed using RStudio and Python programming languages to ensure a comprehensive statistical evaluation. Descriptive statistics, including frequencies, percentages, means, and standard deviations, were calculated to describe participant characteristics and study variables at baseline during the Ramadan period and after. Given the paired nature of the data collected before and after Ramadan from the same participants, paired sample analyses were employed to examine changes in diabetes self-efficacy, self-management behaviors, and self-care activities. Paired sample t-tests were conducted to compare mean scores of continuous variables between the pre-Ramadan and post-Ramadan assessments. The normality of data distribution was assessed using the Shapiro–Wilk test, and when assumptions for parametric tests were not met, the Wilcoxon signed-rank test was used as a non-parametric alternative for paired comparisons.

Pearson correlation coefficients were calculated to examine relationships between continuous variables within each period, while Spearman’s rank correlation was used for non-normally distributed data. Missing data patterns were analyzed, and appropriate methods, listwise deletion, were applied based on the missing data mechanism. Statistical significance was set at *p* < 0.05 for all analyses. Confidence intervals were calculated at the 95% level to provide additional information about the precision of estimates.

## 3 Results

### 3.1 Demographic and clinical characteristics of the study participants

A total of 162 pregnant women with diabetes participated in the study (Table 1). The average age of participants was 34 years (SD = 6). Most women had completed at least secondary education (66.7%), with a smaller proportion having higher education (9.3%) or no formal education (1.9%). Over half of the participants were homemakers (53.7%), followed by full-time employees (31.5%). The vast majority resided in urban areas (88.3%). Regarding pregnancy order, approximately 28% were experiencing their first pregnancy, while 14.2% had more than five previous pregnancies. Most participants received antenatal care from government health centers (61.7%), while 20.4% attended governmental maternity hospitals and 17.9% visited private clinics. A notable majority had no history of diabetes prior to pregnancy (71.6%), though 87% were diagnosed with diabetes either during the current or previous pregnancies.

**Table 1:** Demographic and clinical characteristics of study participants.

Variables		*N* (%)
Age mean (SD)		34 (6)
Education level	No formal education	3 (1.9%)
	Basic education	36 (22.2%)
	Secondary education	108 (66.7%)
	Higher education	15 (9.3%)
Employment status	Full-time employee	51 (31.5%)
	Part-time employee	12 (7.4%)
	Housewife	87 (53.7%)
	Student	12 (7.4%)
Residence	Urban (city/province)	143 (88.3%)
	Rural (village/hajra)	19 (11.7%)
Pregnancy order	First pregnancy	45 (27.8%)
	Second pregnancy	16 (9.9%)
	Third pregnancy	30 (18.5%)
	Fourth pregnancy	13 (8%)
	Fifth pregnancy	35 (21.6%)
	More than fifth	23 (14.2%)
Follow-up clinic	Government health centers	100 (61.7%)
	Private clinics	29 (17.9%)
	Maternity hospitals (Governmental)	33 (20.4%)
Diabetes before pregnancy	No	116 (71.6%)
	Yes	46 (28.4%)
Ever diagnosed with DM during this pregnancy or any previous pregnancies	No	21 (13%)
	Yes	141 (87%)

### 3.2 Self-efficacy and self-care scores during and after Ramadan

Self-efficacy scores remained relatively stable during and after Ramadan, with a mean of 4.8 (SD = 2.2) during Ramadan and 4.9 (SD = 2.1) post-Ramadan (Table 2). This slight increase was not statistically significant (*p* = 0.2). In contrast, self-care activity scores decreased significantly after Ramadan, dropping from 32.5 (SD = 18.1) during Ramadan to 29.8 (SD = 17.2) post-Ramadan (mean change = −2.7, *p* = 0.001).

**Table 2:** Comparison of self-efficacy and self-care scores during and post-Ramadan.

Variables	During Ramadan	Post-Ramadan	Difference	*p*-value*^1^*
	Mean ± SD	Mean ± SD	Mean change	
Self-efficacy score	4.8 ± 2.2	4.9 ± 2.1	+ 0.1	0.2
Self-care activities score	32.5 ± 18.1	29.8 ± 17.2	− 2.7	0.001*

^1^The paired-samples *t* test was used, *Statistically significant *p*-value. Bold values indicate statistically significant results (*p* < 0.05).

### 3.3 HbA1c levels during and after Ramadan

**Figure 1:**
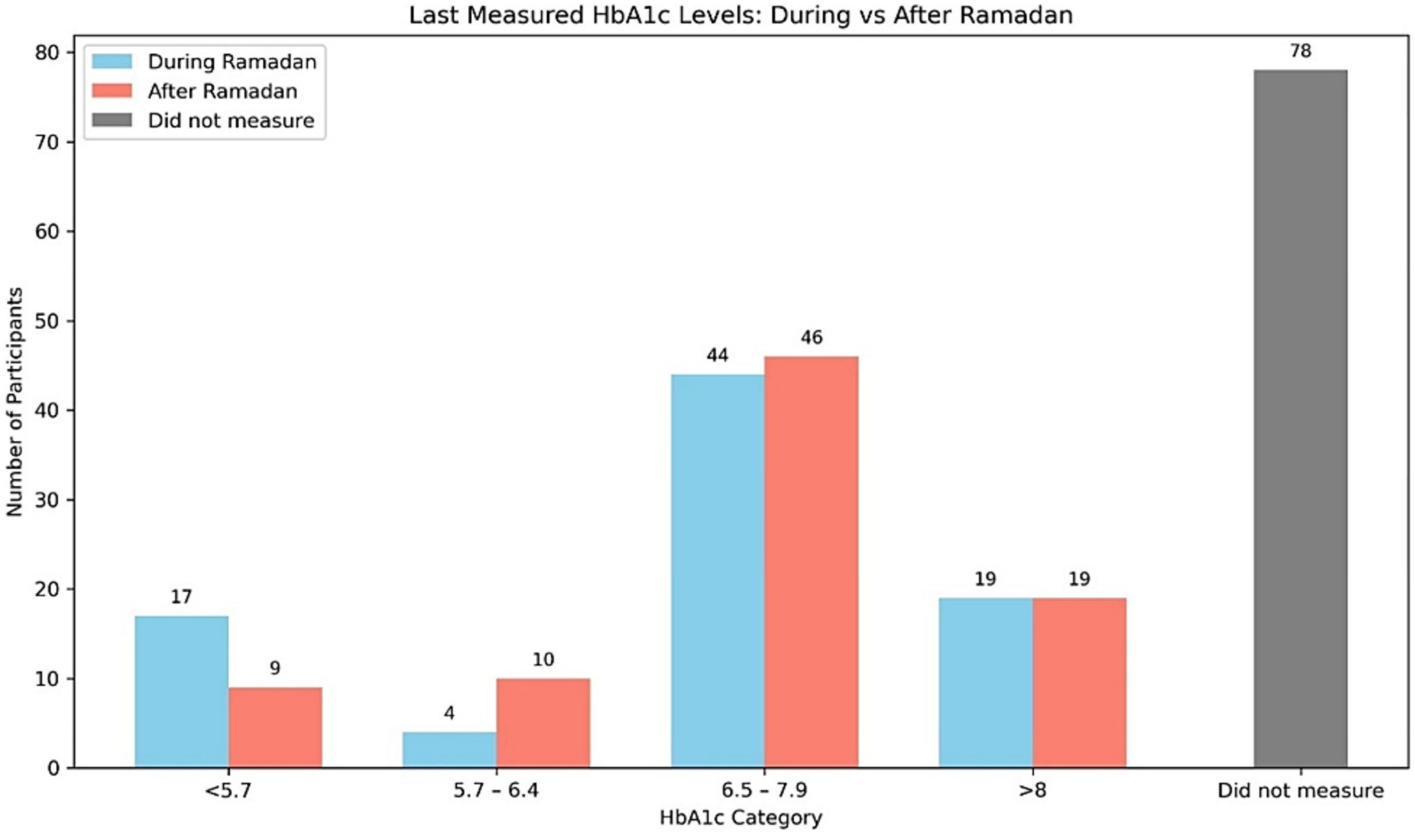
Comparison of last measured HbA1c levels during and after Ramadan among participants.

Figure 1 illustrates the distribution of participants’ last measured HbA1c levels during and after Ramadan. The most common HbA1c category was 6.5–7.9% in both periods, reported by 44 participants during Ramadan and 46 after. There was a slight decrease in the number of participants with HbA1c < 5.7% after Ramadan (from 17 to 9) and a slight increase in those within the 5.7–6.4% category (from 4 to 10). The number of participants with HbA1c > 8% remained unchanged (*n* = 19) in both periods. Notably, a substantial portion of the sample (*n* = 78) did not measure their HbA1c levels.

### 3.4 Diabetes management methods during Ramadan

**Figure 2:**
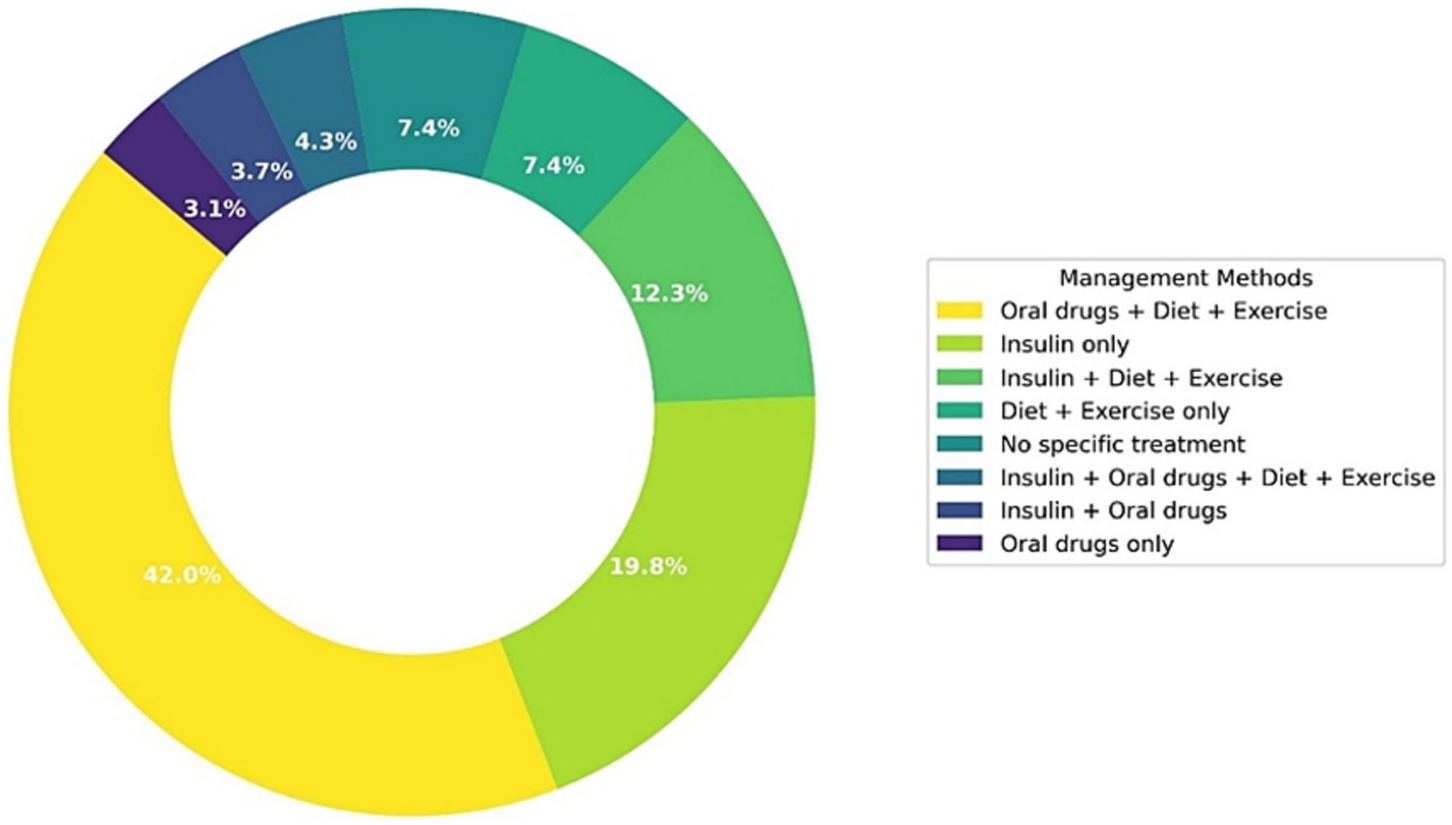
Distribution of diabetes management methods among pregnant women during Ramadan.

Figure 2 shows the distribution of diabetes management strategies used by participants during Ramadan. The most frequently reported method was a combination of oral drugs, diet, and exercise, used by 42% of participants. This was followed by insulin-only management (19.8%) and insulin combined with diet and exercise (12.3%). Smaller proportions reported using only diet and exercise (7.4%) or no specific treatment (7.4%). Other less common regimens included insulin with oral drugs and lifestyle modifications (4.3%), insulin with oral drugs only (3.7%), and oral drugs only (3.1%).

### 3.5 Role of preparation and guidance

Women who received medical education about diabetes before Ramadan demonstrated significantly higher self-care activity scores (35.4 vs. 17.8, *p* < 0.001), although no significant difference in self-efficacy was observed (*p* = 0.60) (Table 3). Participants who prepared for Ramadan by fasting in advance reported significantly higher self-efficacy scores (6.2 vs. 4.5, *p* = 0.001), suggesting that proactive fasting preparation may boost confidence in managing diabetes. However, self-care scores did not differ significantly between those who prepared and those who did not (*p* = 0.44). Those who were advised not to fast had significantly lower self-efficacy scores (4.3 vs. 5.2, *p* = 0.012), although their self-care activity scores were not significantly different (*p* = 0.3).

**Table 3:** Comparison of self-efficacy and self-care scores during Ramadan regarding medical education, fasting behaviors, and medical advice.

Variables		Self-efficacy*^1^*	*p*-value	Self-care*^1^*	*p*-value
Did you receive any medical education about diabetes before Ramadan?	No	4.6 (1.7)	0.60	17.8 (15)	<0.001*
	Yes	4.8 (2.3)		35.4 (17.2)	
Did you try fasting for several days before Ramadan to get used to it?	No	4.5 (2.2)	0.001*	33 (17.9)	0.44
	Yes	6.2 (2)		29.9 (18.9)	
Were you advised not to fast during Ramadan?	No	5.2 (2)	0.012*	33.8 (18.9)	0.3
	Yes	4.3 (2.4)		31 (17)	

^1^Mean score (Standard deviation), *Statistically significant *p*-value. Bold values indicate statistically significant results (*p* < 0.05).

### 3.6 Blood glucose monitoring and glycemic control

There were no significant differences in the frequency of daily blood glucose monitoring before and after Ramadan (*p* = 0.8) (Table 4). Most participants monitored their blood sugar levels three to four times per day, with 22.2% checking three times and 40.7% checking four times during Ramadan, and 20.4 and 42.6% doing so after Ramadan, respectively. However, perceived glycemic control showed significant improvement after Ramadan. The proportion of participants reporting difficulty in controlling their blood sugar decreased from 72.8% during Ramadan to 65.4% post-Ramadan (*p* = 0.001). Additionally, fewer women reported a change in their ability to manage blood glucose after Ramadan (41.4%) compared to during Ramadan (51.2%), reflecting greater stability in glycemic control (*p* = 0.020).

**Table 4:** Blood glucose monitoring and glycemic control during and after Ramadan.

Variables		During Ramadan		After Ramadan		*p*-value*^1^*
		*n*	Column %	*n*	Column %	
How many times did you measure your blood sugar per day during Ramadan?	None	21	13.0%	22	13.6%	0.8
	1 time	11	6.8%	10	6.2%	
	2 times	21	13%	21	13%	
	3 times	36	22.2%	33	20.4%	
	4 times	66	40.7%	69	42.6%	
	5 + times	7	4.3%	7	4.3%	
Do you find it difficult to control your blood sugar?	No	44	27.2%	56	34.6%	0.001*
	Yes	118	72.8%	106	65.4%	
Has your ability to control blood sugar changed during Ramadan?	No	79	48.8%	95	58.6%	0.020*
	Yes	83	51.2%	67	41.4%	

^1^The Wilcoxon signed-rank test and McNemar Test were used, *Statistically significant *p*-value. Bold values indicate statistically significant results (*p* < 0.05).

### 3.7 Reasons for breaking the fast during Ramadan

**Figure 3:**
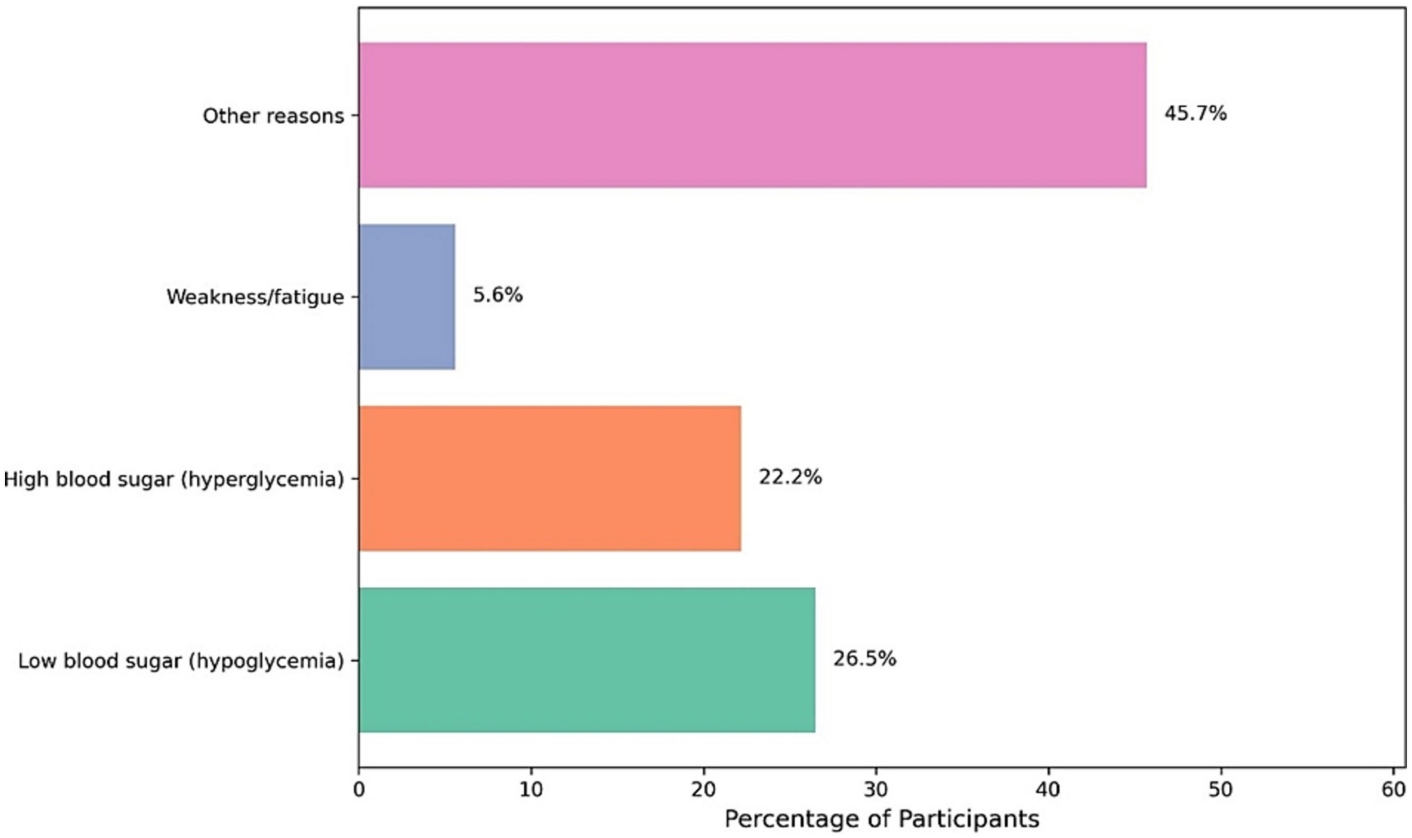
Reasons reported for breaking the fast among diabetic pregnant women during Ramadan.

Figure 3 presents the self-reported reasons for breaking the fast among pregnant women with diabetes during Ramadan. The most commonly cited reason was categorized as “other reasons,” accounting for 45.7% of responses. Hypoglycemia (low blood sugar) was the second most frequent cause, reported by 26.5% of participants, followed by hyperglycaemia (high blood sugar) at 22.2%. Weakness or fatigue was the least reported reason, noted by only 5.6% of the women.

### 3.8 Correlations among key factors influencing diabetes management

**Figure 4:**
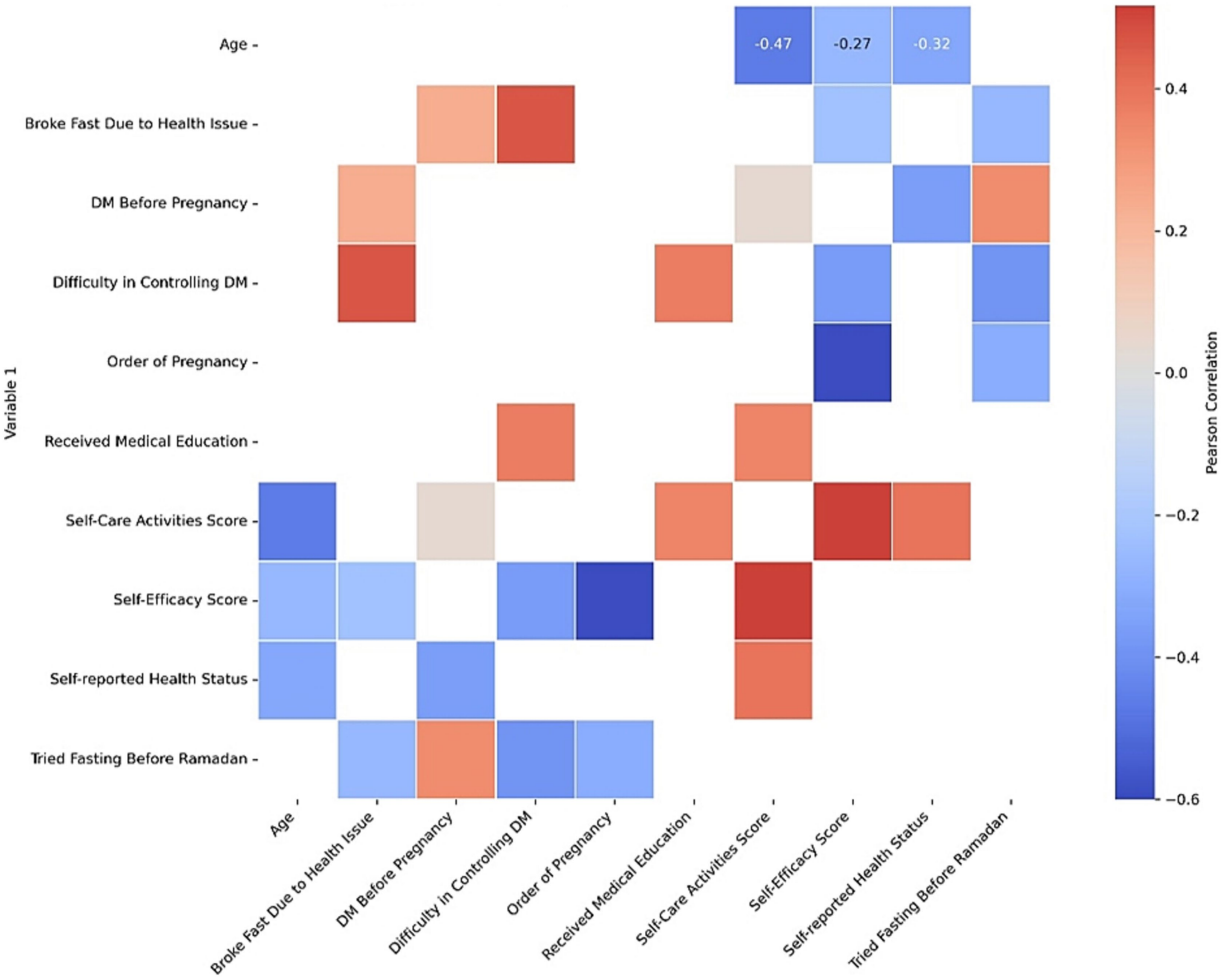
Correlation heatmap of factors influencing diabetes management during Ramadan.

A bivariate correlation analysis revealed several significant associations among key variables influencing diabetes management during and after Ramadan among pregnant women (Figure 4). Age showed a moderate negative correlation with both self-efficacy scores (*r* = −0.268, *p* < 0.01) and self-care activities (*r* = −0.469, *p* < 0.001), indicating that older participants tended to have lower scores in both domains. Self-efficacy was positively associated with self-care activities (*r* = 0.517, *p* < 0.001) but negatively correlated with order of pregnancy (*r* = −0.600, *p* < 0.001), suggesting diminishing confidence with higher parity. Self-care activities were also positively related to receiving medical education (*r* = 0.359, *p* < 0.001) and perceived health status (*r* = 0.395, *p* < 0.001). Difficulty in controlling diabetes was positively correlated with breaking the fast due to health issues (*r* = 0.471, *p* < 0.001) and receiving prior medical education (*r* = 0.376, *p* < 0.001), while it was negatively associated with self-efficacy (*r* = −0.366, *p* < 0.001) and trial fasting before Ramadan (*r* = −0.388, *p* < 0.001). These findings underscore the multifaceted influence of education, self-perception, and previous experience on glycemic control and fasting behaviors.

## 4 Discussion

Our findings shed new light on how pregnant women with diabetes negotiate the demands of Ramadan fasting and the weeks that follow. Although overall confidence in self-management remained steady from the fasting to the post-Ramadan period, we observed a meaningful decline in actual self-care activities once the month concluded. This discrepancy between the sustained sense of self-efficacy and the decline in self-care behaviors reflects findings from previous narrative reviews on diabetes management during Ramadan (2, 4). These reviews highlight that possessing knowledge and confidence alone is not sufficient to maintain long-term behavioral changes once the fasting period concludes. It appears that the structure and reminders inherent to the Ramadan routine help women maintain self-care behaviors during the holy month, but that these supports dissipate once normal schedules resume.

The observed shifts in HbA1c categories (e.g., fewer participants in the <5.7% range post-Ramadan) likely reflect short-term metabolic adaptations to fasting rather than clinically significant deterioration in glycemic control. While statistically significant, these changes—measured within 1 month post-Ramadan—fall within the biological variability of HbA1c and may not indicate long-term risk (13). Notably, similar transient patterns have been reported in pregnant cohorts fasting during Ramadan, where metabolic stress during fasting was followed by stabilization within 6–8 weeks. This pattern aligns with clinical trials demonstrating that newer agents such as GLP-1 agonists and DPP-4 inhibitors confer better short-term control during fasting but require ongoing reinforcement to preserve gains over time (14). In our cohort, nearly half did not obtain post-Ramadan HbA1c measurements, underscoring the need for systems that facilitate follow-up testing. Future studies with extended follow-up could clarify whether these changes persist beyond the immediate recovery period.

Women who received pre-Ramadan medical education in our sample exhibited significantly higher self-care scores, mirroring results from the Egyptian structured-education trial in Beni Suef, where targeted counseling reduced hypoglycemic events by nearly a third and boosted monitoring uptake (7). Likewise, those who fasted in advance reported greater confidence, a finding that supports the practice-fasting recommendation found in international guidelines (15–17). Taken together, these observations reinforce the central role of timely, pregnancy-specific education in fostering both the skills and the habits necessary for safe fasting (18).

Notably, our findings must be interpreted within the cultural and religious context of Ramadan observance. Many pregnant women in our study chose to fast despite medical exemptions, reflecting the deep spiritual significance of Ramadan and societal expectations surrounding participation. This aligns with qualitative studies showing that Muslim women often weigh religious devotion against medical advice, with some viewing fasting as an act of faith that supersedes health risks (19, 20). Future interventions could bridge this gap by incorporating faith-leader engagement and framing medical recommendations within Islamic ethical frameworks.

Despite frequent blood glucose monitoring, with most participants checking levels three to four times daily, perceived difficulty in glycemic control fell after Ramadan. This may reflect a growing familiarity with self-adjustment of therapy or simply relief once the fasting constraints lift (21). It also resonates with survey data from Saudi and Pakistani adults with diabetes showing that although hypoglycemia remains common during Ramadan, most report stable control by the end of the month (3). In our pregnant cohort, hypoglycemia accounted for only a quarter of fast-breaking events; “other” reasons predominated, suggesting that physical discomfort or non-glycemic symptoms may drive early termination of fasts as much as metabolic alarms (15, 22).

The inverse correlations we observed between age, parity and both self-efficacy and self-care warrant particular attention. Older women and those with multiple prior pregnancies reported lower confidence and fewer self-care activities, echoing qualitative work among pregnant Muslim women in London that documented greater reluctance to fast and more reliance on religious counsel when hypoglycemia history is present. It seems that cumulative life experience does not necessarily translate into smoother diabetes management under fasting conditions; in fact, those responsibilities may erode the mental bandwidth available for rigorous self-care.

Finally, the predominance of combination therapy, particularly oral agents plus lifestyle modification, underscores the complex pharmacological landscape that pregnant women navigate. Our finding that nearly half the sample used oral drugs with diet and exercise dovetails with guideline recommendations to tailor regimens according to individual risk, but it also highlights the persistent preference for non-insulin approaches despite recognized safety advantages of insulin during fasting (2, 5). This suggests that efforts to optimize medication counseling must address patient preferences and cultural considerations alongside clinical evidence.

Our study confirms that sustained, culturally appropriate education and close follow-up are essential to translate the heightened vigilance of Ramadan into long-term self-care habits. By aligning pre-Ramadan assessment, medication adjustment, and collaborative support with the lived realities of pregnant women, healthcare teams can help preserve the gains achieved during the holy month and foster enduring improvements in glycemic control.

### 4.1 Limitations

This study has several limitations. The sample size was relatively small, particularly for pregnant participants, which may limit the generalizability of findings. Self-reported data on glycemic control and fasting behavior may be subject to recall bias or social desirability bias. Additionally, the lack of continuous glucose monitoring limited the ability to capture real-time fluctuations in blood glucose. Post-Ramadan follow-up was incomplete for a significant portion of participants, affecting the assessment of long-term outcomes. Cultural and healthcare system differences between regions may also influence results, and findings may not be universally applicable without considering local practices and healthcare access. A notable proportion of participants (*n* = 78) did not complete HbA1c testing during or after Ramadan, which may limit the generalizability of glycemic control findings. While reminders were provided, barriers such as logistical challenges (e.g., travel to testing facilities), financial constraints, or perceived stability of glucose levels may have contributed to non-compliance. This highlights a potential gap in real-world adherence to monitoring protocols among pregnant women with diabetes, particularly in such settings. Future studies could mitigate this by incorporating point-of-care HbA1c testing during clinic visits or using telehealth follow-ups to improve accessibility. While our findings demonstrate a positive association between medical education and improved outcomes, we recognize the potential for self-selection bias. Participants who actively sought or were more receptive to diabetes education may have been inherently more motivated to engage in self-care behaviors, which could have influenced their outcomes independently of the educational intervention.

## 5 Conclusion

This study highlights the complex interplay of medical, behavioral, and educational factors influencing diabetes management among pregnant women during and after Ramadan. While self-efficacy remained stable, a decline in self-care activities post-Ramadan suggests the need for sustained support beyond the fasting month. Medical education, pre-Ramadan preparation, and tailored advice were positively associated with better outcomes. These findings emphasize the importance of culturally sensitive, pregnancy-specific interventions to ensure safe fasting practices. Healthcare providers should prioritize individualized care plans and ongoing education to support this high-risk group in achieving optimal glycemic control while respecting their religious practices.

## Data Availability

The original contributions presented in the study are included in the article/supplementary material, further inquiries can be directed to the corresponding authors.
